# The depolymerase activity of MCAK shows a graded response to Aurora B kinase phosphorylation through allosteric regulation

**DOI:** 10.1242/jcs.228353

**Published:** 2019-01-14

**Authors:** Toni McHugh, Juan Zou, Vladimir A. Volkov, Aurélie Bertin, Sandeep K. Talapatra, Juri Rappsilber, Marileen Dogterom, Julie P. I. Welburn

**Affiliations:** 1 Wellcome Trust Centre for Cell Biology, School of Biological Sciences, University of Edinburgh, Edinburgh EH9 3BF, UK; 2 Department of Bionanoscience, Faculty of Applied Sciences, Delft University of Technology, Delft 2629, The Netherlands; 3 Laboratoire Physico Chimie Curie, Institut Curie, PSL Research University, CNRS UMR168, 75005 Paris, France; 4 Sorbonne Universités, UPMC University Paris 06, 75005 Paris, France; 5 Chair of Bioanalytics, Institute of Biotechnology, Technische Universität Berlin, Berlin 10623, Germany

**Keywords:** MCAK, Aurora B, Microtubules, Phosphorylation

## Abstract

Kinesin-13 motors regulate precise microtubule dynamics and limit microtubule length throughout metazoans by depolymerizing microtubule ends. Recently, the kinesin-13 motor family member MCAK (also known Kif2C) has been proposed to undergo large conformational changes during its catalytic cycle, as it switches from being in solution to being bound to microtubules. Here, we reveal that MCAK has a compact conformation in solution through crosslinking and electron microscopy experiments. When MCAK is bound to the microtubule ends, it adopts an extended conformation with the N-terminus and neck region of MCAK interacting with the microtubule. Interestingly, the region of MCAK that interacts with the microtubule is the region phosphorylated by Aurora B and contains an end binding (EB) protein-binding motif. The level of phosphorylation of the N-terminus results in a graded microtubule depolymerase activity. Here, we show that the N-terminus of MCAK forms a platform to integrate Aurora B kinase downstream signals and in response fine-tunes its depolymerase activity during mitosis. We propose that this allosteric control mechanism allows decoupling of the N-terminus from the motor domain of MCAK to allow MCAK depolymerase activity at kinetochores.

## Abstract (precis)

**Summary:** The kinesin-13 MCAK has a compact conformation in solution but is extended when bound to microtubules. Aurora B phosphorylation of MCAK inhibits depolymerase activity by disrupting its extended conformation.

## INTRODUCTION

Regulation of microtubule length is key throughout eukaryotes. Notably, the kinesin-13 family members are potent microtubule depolymerases, which destabilize microtubule ends to promote catastrophe (Walczak et al., 2013). Kinesin-13 members play a critical role during mitosis as they are involved in achieving correct spindle assembly and positioning and accurate kinetochore–microtubule attachments to prevent genomic instability. The tight regulation of microtubule depolymerases is critical to the control of microtubule length spatially and temporally.

The most potent kinesin-13 depolymerase is Kif2C, also termed MCAK. The activity of MCAK is inhibited by Aurora B kinase, through phosphorylation on multiple amino acids within its N-terminus (Andrews et al., 2004; Lan et al., 2004). These sites are adjacent to the ‘SxIP’, end binding (EB) protein-binding motif and the neck linker region. EB proteins (also known as MAPRE proteins) bind MCAK and recruits it to microtubule plus ends (Mennella et al., 2005; Montenegro Gouveia et al., 2010; Moore et al., 2005). Phosphorylation of residues close to the EB-binding motif prevents MCAK association with EB proteins and subsequent MCAK recruitment to the plus ends of microtubules (Honnappa et al., 2009; Moore et al., 2005). Aurora B kinase phosphorylation of the N-terminus of MCAK also inhibits its microtubule depolymerase activity *in vitro* in the absence of EB proteins (Lan et al., 2004), although the mechanism is unclear. Paradoxically, the recruitment of MCAK to centromere-tethered Sgo2, where MCAK controls inter-kinetochore stretching and chromosome alignment, requires Aurora B kinase activity (Huang et al., 2007; Rattani et al., 2013; Tanno et al., 2010). Thus, Aurora B kinase may play a role in fine-tuning the depolymerase activity of MCAK at centromeres rather than fully inhibiting MCAK.

Most *in vitro* studies on the mechanism of MCAK focus on the monomeric motor domain of MCAK. However, full-length MCAK acts as a dimer *in vivo* (Maney et al., 2001). To understand how MCAK destabilizes microtubules in mitosis, it is critical to examine how full-length MCAK interacts with the microtubule and how Aurora B phosphorylation controls the intrinsic activity of MCAK at the molecular level. However, the structure of full-length MCAK and the molecular mechanism for MCAK regulation remains unclear. To gain insights into the structure of full-length MCAK and its regulation by phosphorylation, we performed biochemistry and crosslinking/mass spectrometry experiments. We demonstrate that full-length MCAK has a compact conformation in solution and becomes extended when bound to microtubules. Aurora B phosphorylation interferes with the extended conformation of MCAK on microtubules. Overall we show that rather than just inhibiting it, Aurora B fine-tunes the activity of MCAK activity to provide graded levels of microtubule depolymerase activity in the cell.

## RESULTS AND DISCUSSION

### Full-length dimeric MCAK has a compact structure in solution

**Fig. 1.**
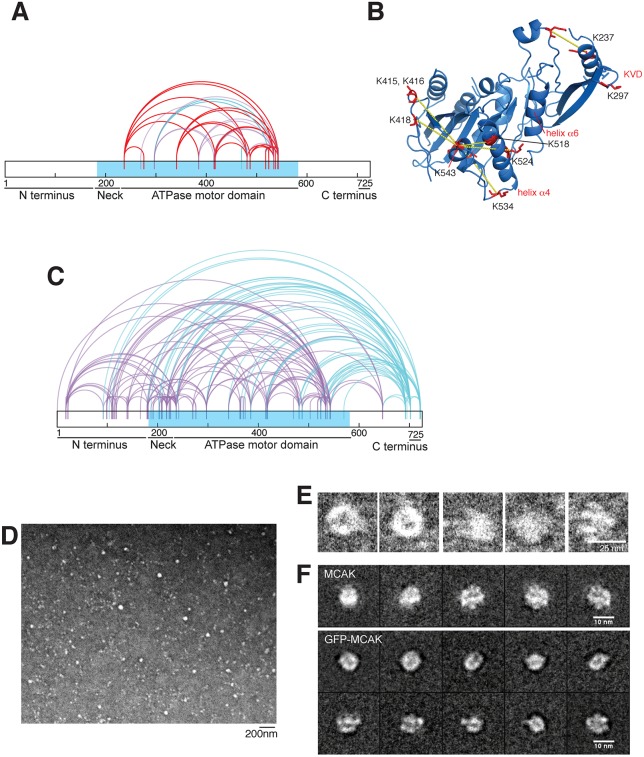
**MCAK has a compact conformation in solution.** (A) Crosslinks for amino acids in the MCAK motor domain crystal structure (PDB 4UBF, 2HEH) within 27Å in the monomer (red), between two monomers in the asymmetric unit (blue) or not present (purple). (B) MCAK motor domain structure (PDB 2HEH) showing crosslinks (dashed lines) and lysine amino acids involved (red). (C) Crosslinking pattern of full-length MCAK highlighting the motor domain (blue) and crosslinks involving the far C-terminus (purple). (D) Negative stain EM micrograph of MCAK particles. (E) MCAK particles picked for image analysis and classification. (F) Class averages of negative-stained MCAK and MCAK–GFP.

Early studies using rotary shadowing electron microscopy (EM) on the kinesin-13 Kif2a showed that kinesin-13 family proteins have a globular structure in solution (Noda et al., 1995). Given the high sequence conservation between Kif2a and MCAK, we hypothesized that MCAK would also form a compact structure in solution. To test whether long-range interactions stabilize full-length MCAK to form a compact motor, we analyzed the 3D structure of human MCAK in solution using crosslinking and mass spectrometry. We first used the zero-length crosslinker EDC/NHS on MCAK and obtained no crosslinks. However, by using the BS3 crosslinker, we were able to successfully crosslink MCAK (Fig. S1A). We isolated a species with a higher molecular mass of 160 kDa, corresponding to dimeric MCAK, from a denaturing gel (Fig. S1A). We then analyzed the crosslinked peptides and identified 160 crosslinks (Fig. S1B,C). To validate them, we examined whether the crosslinks obtained within the motor domain were compatible with the crystal structure of the MCAK motor domain (PDB 2HEH). The majority of crosslinks obtained correspond to lysine residues within 27 Å of each other in the MCAK motor domain, corresponding to the maximum length of BS3 (Fig. 1A,B). A few crosslinks identified could be due to the dimeric arrangement of MCAK in the presence of the C-terminus (Talapatra et al., 2015). Overall, our data confirmed that the 3D crosslinking/mass spectrometry was highly specific for the native conformation of MCAK in solution.

We next examined the crosslinks present in MCAK between the motor and non-motor regions. MCAK is dimeric; thus, it is not possible to distinguish whether the crosslinks are intermolecular or intramolecular. We found that the MCAK N-terminus crosslinks with the C-terminus (Fig. 1C; Fig. S1C). We also identified crosslinks between the N- and C-termini of MCAK and the motor domain, suggesting the non-motor regions ‘sample’ conformations where they are in close proximity to the motor domain. In particular, we observed multiple crosslinks between the far C-terminus of dimeric MCAK and the motor domain, indicating that the C-terminus of MCAK is in close proximity with its motor domain in the context of full-length MCAK (Fig. 1C). These data further support the model that the C-terminus of MCAK binds to the N-terminus and motor domain in solution (Maney et al., 2001; Moore et al., 2005; Talapatra et al., 2015; Zong et al., 2016). Interestingly, the MCAK C-terminus crosslinked with residues in a region that encompasses the α4 helix and L12 loop (522–541), which contribute towards the switch II region and the microtubule-binding site. This is in good agreement with the fact that the C-terminus of MCAK and the microtubule compete for the microtubule-binding site of MCAK (Talapatra et al., 2015). Overall, this crosslinking approach does not allow us to dissect the contribution of each monomer to the dimeric MCAK organization but points to a compact conformation of MCAK, where the N- and C-termini of MCAK interact with the motor domain and with each other extensively in solution to form a compact dimer, unlike more-extended conventional kinesins.

To gain further insights into the structure of full-length MCAK, we examined full-length dimeric MCAK through negative stain EM (Fig. 1D,E). We observed that MCAK has a compact structure, similar to that of Kif2a (Hirokawa, 1998). We then performed 2D class averages of MCAK and GFP-tagged MCAK. A set of representative classes obtained after two successive rounds of 2D alignment and classification are displayed in Fig. 1F. U-shaped structures or opened rings are obtained, corresponding to different views of the protein. The U-shaped view is 9.5 nm long and 5.5 nm wide. The visualized rings are ∼9.5 nm in diameter. The folding and compactness of MCAK is not impaired by the presence of a GFP tag at its C-terminus. By comparison with a similar analysis performed on MCAK deprived of GFP, we cannot detect any significant difference, implying that the GFP tag might be mobile and not visualized in the classes. Although we could not define the motor domain positions in the class averages, we can conclude that full-length MCAK has a compact conformation in solution.

### Aurora B phosphorylation modestly affects MCAK conformation in solution

Aurora B phosphorylates the N-terminus of MCAK to reduce its activity and disrupts its interaction with EB proteins (Andrews et al., 2004; Honnappa et al., 2009; Knowlton et al., 2006; Ohi et al., 2004) (Fig. S1D). We next examined whether Aurora B phosphorylation of MCAK induces conformational changes that could inhibit MCAK. We generated an Aurora B phosphomimetic MCAK mutant for six serine residues (MCAK_S6E_) to ensure the sample was fully modified on the residues that are phosphorylated *in vivo.* We mutated residues Ser95, Ser109, Ser111, Ser115, Ser166 and Ser192 to glutamate to mimic constitutive phosphorylation. Through quantitative mass spectrometry, we did not find unique crosslinks belonging to either MCAK or MCAK_S6E_. We found 130 common crosslinks in both MCAK and MCAK_S6E_ with peak intensity ratios of MCAK:MCAK_S6E_ of 1.77–0.30 (Fig. S1E), indicating they have a similar conformation in solution. The crosslinks with significant intensity ratios changes are listed in Table 1 (*t*-test, *P*<0.05). The crosslink pairs containing peptide I^100^PAPKESLR^108^, had significantly decreased intensity in MCAK_S6E_ compared to MCAK. This peptide is in the region phosphorylated by Aurora B and makes crosslinks with both the motor domain and C-terminus (Fig. S1D). Thus, our data support that Aurora B-mediated phosphorylation of the MCAK N-terminus slightly opens the compact conformation of MCAK and diminishes the N-terminus-motor and C-terminus interaction, as previously reported using FRET (Ems-McClung et al., 2013). However, Aurora B phosphorylation of the MCAK N-terminus does not induce any major changes in the compact conformation of MCAK in solution.

**Table 1.**
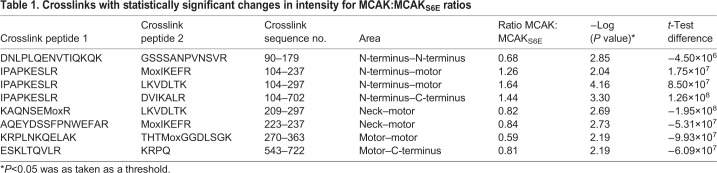
**Crosslinks with statistically significant changes in intensity for MCAK:MCAK_S6E_ ratios**

### The N-terminus of MCAK interacts with the microtubule

**Fig. 2.**
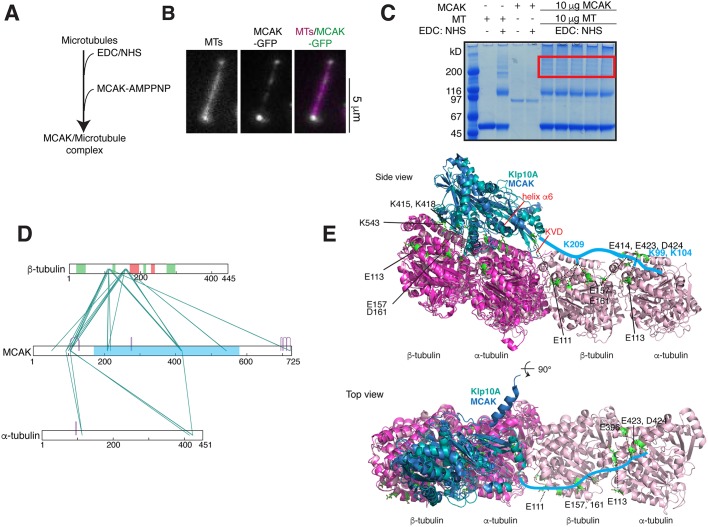
**MCAK has an extended conformation on microtubules.** (A) Experimental setup for crosslinking MCAK to microtubule ends. Microtubules were pre-activated with EDC/sulfo-NHS, followed by the addition of MCAK and AMP-PNP. (B) Representative image of a microtubule bound to MCAK–GFP in the presence of AMPPMP *in vitro*. (C) SDS-PAGE Coomassie-stained gel showing MCAK–tubulin crosslinking with EDC/sulfo-NHS. The region selected for trypsin digestion and peptide extraction is boxed in red. (D) Linkage map showing sequence positions of the crosslinked residue pairs between MCAK and porcine α- and β-tubulin. Regions of β-tubulin involved in longitudinal (coral) and lateral (green) contacts and the MCAK motor domain (sky blue) are highlighted. (E) Structural model of MCAK- and Klp10A-binding microtubules in cartoon representation. The MCAK–tubulin crystal structure (blue:magenta, PDB 5MIO) is overlaid with the cryo-EM structure of the kinesin-13 family protein Klp10A, bound to microtubules (cyan, PDB 6B0C). Tubulin dimers from the structure of stathmin–tubulin–TTL are overlaid in pink (PDB 4IIJ). MCAK–tubulin crosslinks are highlighted green in stick representation. The N-terminal extension of MCAK is drawn in light blue.

To further examine how Aurora B might affect the interaction between the microtubule and MCAK to inhibit MCAK, we mapped the regions of MCAK that are proximal to microtubule ends. To generate a MCAK–microtubule complex that would mimic the MCAK conformation at microtubule ends, we incubated MCAK with AMP-PNP in the presence of taxol-stabilized microtubules (Andrews et al., 2004; Lan et al., 2004; Zhang et al., 2008) (Fig. 2A,B). We used the EDC/NHS crosslinker to identify the amino acids involved in the MCAK–microtubule interaction (Fig. 2C). Crosslinks were detected between MCAK and the surface of α- and β-tubulin (Fig. 2D). We found that residues 415, 418 and 542 in the β5 sheet (close to loop L8) and loop L12 crosslink with β-tubulin, indicating they are close to the microtubule core. Our crosslinking data confirm that MCAK binds to the microtubule core directly, rather than to the acidic tail of tubulin. Surprisingly, we also observed multiple crosslinks between the N-terminus of MCAK and the microtubule. Residues Lys92, Lys99, Lys104 and Ser106, close to the EB-binding motif and the Aurora B phosphorylation sites, crosslinked to Glu113, Asp414, Glu423 and Asp424 in α-tubulin. These residues and Lys209 in the neck linker region of MCAK also crosslinked to the acidic patches contributed by Glu108, Glu111, Asp114, Glu157, Glu158 and Asp161 in β-tubulin. Using our crosslinking data and crystal structures of MCAK, kinesin-1 and stathmin bound to two tubulin dimers (mimicking microtubules), we could generate a molecular model of MCAK motor–microtubule (Fig. 2E) (Gigant et al., 2013; Prota et al., 2013). The MCAK motor binds across a tubulin dimer, leaving space for the N-terminus to bind to the longitudinally adjacent tubulin dimer. Our data demonstrate that MCAK adopts an extended conformation when bound to the microtubule and interacts with the microtubule along two tubulin dimers through both its motor domain and its N-terminus.

### Aurora B kinase gradually reduces the affinity and activity of MCAK for microtubules

**Fig. 3.**
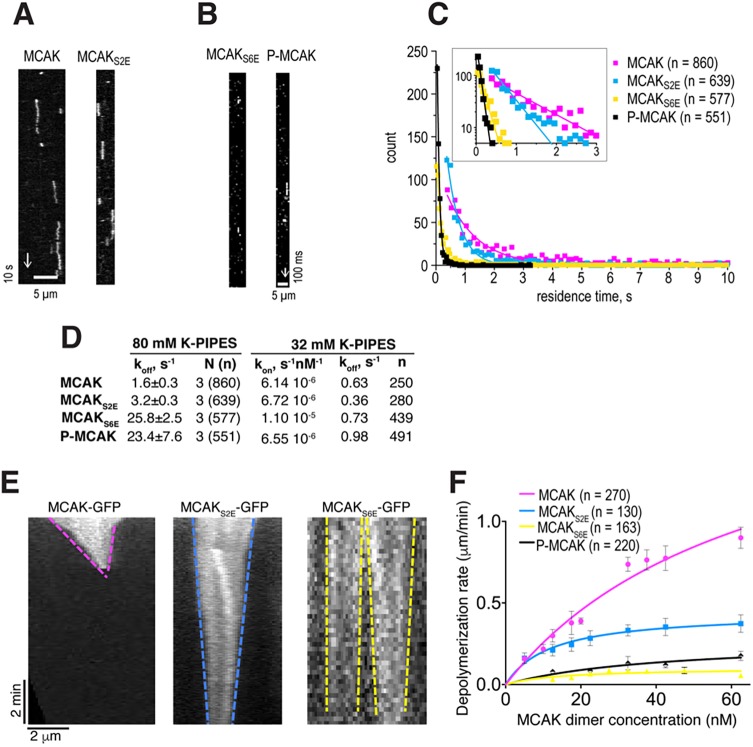
**Aurora B inhibits MCAK by gradually increasing its microtubule off-rate.** (A) Representative kymographs showing single molecules of MCAK–GFP and MCAK_S2E_–GFP diffusing on stable microtubules in 80 mM K-PIPES. (B) Kymographs of binned acquisitions used to quantify residence times of MCAK_S6E_–GFP and phosphorylated MCAK–GFP. (C) Distributions of residence times for MCAK–GFP mutants and Aurora B-phosphorylated MCAK–GFP in 80 mM K-PIPES fitted with single exponential curves. (D) Association and dissociation rates of MCAK–GFP mutants and Aurora B-phosphorylated MCAK–GFP in 80 mM and 32 mM K-PIPES. (E) Representative kymographs of microtubules depolymerizing in the presence of MCAK–GFP mutants (125 nM). (F) Average microtubule depolymerization rates (mean±s.e.m.) for MCAK–GFP mutants and Aurora B-phosphorylated MCAK-GFP, varying concentrations, fitted with modified Hill equations.

The N-terminus and the neck of MCAK, which interact with the microtubule lattice, are phosphorylated on multiple residues by Aurora B to inhibit MCAK activity (Andrews et al., 2004; Lan et al., 2004). Interestingly, these sites lie outside of the motor domain, which is the primary microtubule-binding region and has depolymerase activity. To define how Aurora B mechanistically reduces MCAK activity, we used MCAK–GFP, MCAK_S2E_–GFP, which is mutated in the neck region with Ser192 and Ser166 replaced by glutamate residues, and MCAK_S6E_–GFP, as above (mutated both in the neck and N-terminus). To determine the residency time and affinity of MCAK for microtubules, we imaged single molecules of MCAK–GFP on microtubules in 80 mM K-PIPES (Fig. 3A–C). We found that both MCAK–GFP and MCAK_S2E_–GFP could be observed on the lattice. MCAK–GFP had a slightly longer residency time than MCAK_S2E_–GFP (Fig. 3A,C). MCAK_S6E_–GFP could barely be observed on microtubules, only remaining bound for a very short amount of time (Fig. 3B,C).

To confirm that Aurora B phosphorylation affects the ability of MCAK to bind to microtubules, we phosphorylated MCAK–GFP *in vitro* with Aurora B kinase and imaged it on microtubules (Fig. 3B). Similar to what was seen with MCAK_S6E_-GFP, Aurora B-phosphorylated MCAK very rarely bound to the microtubule and only for very short times, resulting in reduced microtubule residency (Fig. 3C,D). At low ionic strength, MCAK mutants and wild-type microtubule association rates were largely comparable (Fig. 3D; Fig. S2). In 80 mM K-PIPES, MCAK-GFP and MCAK_S2E_-GFP had off-rates of 1.6±0.3 s^−1^ and 3.2±0.3 s^−1^ respectively, while the off-rates for MCAK_S6E_–GFP and Aurora B-phopshorylated MCAK–GFP were significantly and comparably increased to 25.8±2.5 s^−1^, and 23.4±7.6 s^−1^, respectively (mean±s.d.; Fig. 3D). Phosphomimetic mutations in MCAK and Aurora B-phosphorylation of MCAK increased the MCAK off-rates from the microtubules to the same extent in different ionic conditions (Fig. 3D; Fig. S2), indicating that the phosphomimetic mutant recapitulates Aurora B-phosphorylated MCAK *in vitro*. Thus, Aurora B phosphorylates the neck and N-terminus of MCAK to reduce the affinity of MCAK for microtubules.

To test the implications of Aurora B dependent-phosphorylation of the N-terminus and neck of MCAK, we analyzed the microtubule depolymerization activity of GFP-tagged MCAK, MCAK_S2E_ and MCAK_S6E_. In a total internal reflection fluorescence (TIRF) microscopy-based assay, we incubated MCAK–GFP in the presence of Rhodamine-labeled taxol-stabilized microtubules. We then imaged the rate of microtubule depolymerization for MCAK, MCAK_S2E_ and MCAK_S6E_. 62.5 nM dimeric MCAK–GFP rapidly depolymerized microtubules at a rate of 0.90±0.06 µm min^−1^. MCAK_S2E_–GFP displayed an intermediate level of depolymerization activity with a rate of 0.37±0.05 µm min^−1^, while the activity of MCAK_S6E_–GFP was further significantly reduced, with a rate of 0.03±0.01 µm min^−1^ (mean±s.e.m.; Fig. 3E,F). Aurora B-phosphorylated MCAK at the same concentration depolymerized microtubules at 0.18±0.03 µm min^−1^. In all cases, depolymerization occurred at both ends of the microtubules (Fig. 3F).

In conclusion, through EM and crosslinking, we reveal that MCAK has a compact conformation in solution. Our results are in good agreement with FRET data and confirm the X-ray structure of the C-terminus of MCAK bound to its motor domain in solution (Ems-McClung et al., 2013; Talapatra et al., 2015; Zong et al., 2016). In the presence of microtubules, MCAK switches to an extended conformation, where the N-terminus, the neck and the motor domain of MCAK engage with the microtubule core, while the C-terminus is displaced from the motor (Talapatra et al., 2015). The N-terminus of MCAK likely binds a tubulin dimer longitudinally adjacent to that occupied by the motor domain. Recent studies have revealed that the full-length MCAK binds over two tubulin dimers, as does the *Drosophila* homolog Klp10A and human Kif2A (Benoit et al., 2018; Mulder et al., 2009; Ogawa et al., 2017; Trofimova et al., 2018). No electron density is observed for the MCAK N-terminus in the EM class averages, likely due to its largely unstructured properties. The neck linker regions of Klp10A and Kif2a are apparent at the intra-dimer interface of the adjacent tubulin heterodimer (Benoit et al., 2018; Trofimova et al., 2018), in good agreement with our data.

Most work on MCAK points towards the motor domain being the main contributor to the interaction of MCAK with microtubules (Ogawa et al., 2017; Patel et al., 2016; Wang et al., 2017, 2015). Our work reveals that the N-terminus of MCAK plays a major role in stabilizing dimeric MCAK on both the microtubule lattice and at microtubule ends prior to ATP hydrolysis. These results are surprising given the neck–motor domain alone (amino acids 181–583) has a microtubule-binding interface and displays substantial depolymerase activity (Maney et al., 2001). MCAK is dimeric *in vivo*. Thus, one N-terminus could bind to EB proteins to track the growing ends of microtubules while the other engages with the microtubule lattice to trigger microtubule catastrophe. Our results indicate the N-terminus plays an allosteric role in regulating the motor-domain–microtubule interaction or may act to optimally position MCAK within the context of the dimer for catalysis.

During mitosis, Aurora B phosphorylates MCAK to dramatically reduce its depolymerase activity in the vicinity of chromosomes and promote spindle assembly and kinetochore capture. Aurora B acts through two mechanisms: first it phosphorylates the MCAK N-terminus containing the SxIP motif and prevents EB binding (Honnappa et al., 2009). Second, it phosphorylates MCAK on multiple sites to provide a graded level of microtubule depolymerization activity, by modulating the affinity of MCAK for the microtubule lattice. Even a full Aurora B phosphomimetic mutant retains a low microtubule depolymerase activity. This is important because the recruitment of MCAK to the centromere receptor Sgo2 requires Aurora B activity (Huang et al., 2007; Tanno et al., 2010). Centromeric MCAK is phosphorylated to some extent by Aurora B, but still retains modest activity to perform its function (Andrews et al., 2004). At centromeres, MCAK regulates interkinetochore stretch, kinetochore oscillations and error correction of merotelic attachments (Jaqaman et al., 2010; Rattani et al., 2013; Wordeman et al., 2007). The proximity of Aurora B at the centromere may determine the number of sites phosphorylated on MCAK. The extent of MCAK phosphorylation could then provide graded levels of microtubule depolymerase activity depending upon signals integrated by Aurora B at centromeres, such as kinetochore–microtubule attachment status. However, the depolymerase activity of Aurora B-phosphorylated MCAK remains weak. Another possibility is that when the N-terminus of MCAK is bound to its centromeric receptor, the microtubule depolymerase activity of the motor domain is uncoupled from the N-terminus. The allosteric regulation of MCAK is then lost and the motor domain acts alone with restored depolymerase activity, which could explain the role of MCAK in error correction at merotelic attachments. Future work should address how centromere-bound MCAK engages with the kinetochore microtubules to regulate this dynamic interface.

## MATERIALS AND METHODS

### Cloning, expression and purification of MCAK and Aurora B

MCAK and MCAK–GFP constructs were cloned into pFastBac and expressed in Sf9 insect cells. Site-directed mutagenesis was performed using Quikchange mutagenesis kit (Agilent). For Aurora B kinase purification, pEC-K-His-SUMO-SENP2-AuroraB_55-34_ and MCNcs-Spectinomycin-INCENP_835-903_ (supplied by Jeyaprakash Arulanandam, Wellcome Trust Centre for Cell Biology, Edinburgh, UK) were co-expressed in the presence of 50 µg/ml spectinomycin and 50 µg/ml kanamycin in BL21 codon+ cells overnight at 18°C. Protein expression and purification was performed as previously described in (Talapatra et al., 2015). Protein phosphorylation *in vitro* was performed as previously described (Welburn et al., 2010).

### Microtubule depolymerization

Silanized coverslips were prepared as in Bechstedt et al. (2011) except that in place of treatment with Piranha solution, slides were incubated overnight in 12% hydrochloric acid at 50°C. Flow chambers were formed using double-sided sticky tape, a silanized coverslip and a microscopy slide. Flow chambers were 7–8 μl in volume.

All experiments were carried out in BRB80 buffer. Anti-β tubulin antibodies (T7816, Sigma Aldrich) at a 1:10 dilution were first introduced to the chamber. The surface was then blocked with 1% pluronic F-127 (Sigma Aldrich) and Taxol-stabilized 7% Rhodamine-labeled microtubules (Cytoskeleton Inc.) were then bound to the glass surface via the antibodies. The surface was then further blocked with BRB80 buffer containing 1 mg/ml casein (Sigma Aldrich) and 20 μM paclitaxel (Sigma Aldrich). Assay buffer consisted of BRB80 with 1 mM ATP, 0.5 mg/ml casein, 20 μM paclitaxel and an oxygen scavenging system (0.2 mg/ml glucose oxidase, 0.035 mg/ml catalase, 4.5 mg/ml glucose and 140 mM β-mercaptoethanol). Varying concentrations of MCAK, MCAK_S2E_, MCAK_S6E_ and phosphorylated MCAK in assay buffer were introduced to the flow chamber, the chamber was then imaged immediately at 37°C. Imaging was performed on a Zeiss Axio Observer Z1 TIRF microscope using a 100× NA1.46 objective and a Photometrics Evolve Delta EMCCD camera controlled by Zeiss Zen Blue software. Depolymerization assays were performed for up to 10 min at 30 frames per min (fpm) or for MCAK_S6E_ and phosphorylated MCAK and low concentrations of MCAK and MCAK_S2E_ a lower frame rate of 6 fpm was used and imaging was performed for 15 min. Kymographs were produced using ImageJ. Depolymerization rates were measured from these kymographs. Images were stored and vizualized using an OMERO.insight client (OME) (Allan et al., 2012).

### Single-molecule TIRF microscopy

Measurement of MCAK residence time on microtubule lattice was performed as described previously (Volkov et al., 2018). Microtubules were polymerized in MRB80 buffer (80 mM K-PIPES pH 6.9, 1 mM EGTA and 4 mM MgCl_2_) containing 50 µM tubulin (8% digoxigenin-labeled, 3.5% labeled with HyLite-647), 1 mM GTP and 20% glycerol. After 20 min at 37°C, Taxol was added to the final concentration of 25 µM, microtubules were polymerized for 10 min more, and then sedimented in Airfuge (Beckman) for 3 min at 14 psi, and resuspended in 50 µl MRB80 with 40 µM taxol. Coverslips and slides were cleaned using oxygen plasma for 3 min, immediately dipped in a solution of Plus-One Repel Silane (Sigma Aldrich) for 5 min, then sonicated in ethanol and rinsed with water.

Flow chambers were made with silanized slides and coverslips, filled with ∼0.2 µM anti-DIG antibody (11333089001, Roche, Switzerland) and passivated with 1% Tween-20. Then Taxol-stabilized microtubules diluted 1:60 were introduced, unbound material was washed out with MRB80, and MCAK at 5–100 pM was introduced in MRB80 supplemented with 1 mM ATP, 1 mg/ml κ-casein, 40 µM taxol, 4 mM DTT, 0.2 mg/ml catalase, 0.4 mg/ml glucose oxidase and 20 mM glucose. Similarity of the on-rates between MCAK constructs was assayed in the same buffer with reduced ionic strength (32 mM K-PIPES, 0.4 mM EGTA and 1.6 mM MgCl_2_).

MCAK on a microtubule lattice was imaged at 23°C using a Nikon Ti-E microscope (Nikon, Japan) equipped with the perfect focus system (Nikon), a Plan Apo 100×1.45 NA TIRF oil-immersion objective (Nikon), iLas^2^ ring TIRF module (Roper Scientific) and a Evolve 512 EMCCD camera (Roper Scientific, Germany). Images were acquired using MetaMorph 7.8 software (Molecular Devices, San Jose, CA) in stream mode (130 ms per frame at full camera chip, 0.16 µm/pixel). To measure the shorter residence times of MCAK_S6E_ and Aurora B-phosphorylated MCAK in 80 mM K-PIPES, images were binned 8×8 during acquisition, increasing the temporal resolution (to 15 ms per frame), but decreasing spatial resolution (0.86 µm/pixel with an additional 1.5× relay lens).

Kymographs were produced in Fiji (Schindelin et al., 2012) using a reslice operation. Residence times were quantified by measuring the time between the landing and detaching of a single MCAK molecule in the kymograph. Only events longer than three consecutive frames were taken into account. Distributions of residence times were fitted with single exponential decay curves (Origin Pro 9.0) with offset fixed to 0. Off-rate was quantified as 1/τ. To measure on-rate, the total number of landing events was normalized to total observation time, total microtubule length and MCAK concentration as described previously (Zaytsev et al., 2015).

### Protein crosslinking

The mixing ratio of bis(sulfosuccinimidyl)suberate (BS3, Thermo Fisher Scientific) to complex was determined for MCAK using 5 µg aliquots and using a protein-to-crosslinker ratio (w/w) of 1:1, 1:2, 1:3, 1:4, 1:5, 1:6 and 1:7 (Fig. S1). As the best condition, we chose the ratio that was sufficient to convert most of MCAK into a dimeric form as judged by SDS-PAGE analysis. 10 µg MCAK was mixed with 20 µg BS3 dissolved in crosslinking buffer (10 mM HEPES pH 7, 200 mM potassium acetate pH 7.6) and incubated on ice for 1 h. The reaction was stopped by adding 2.5 M ammonium bicarbonate for 20 min on ice. For MCAK–microtubule crosslinking, 10 µg microtubules were preactivated using a mixture of EDC [1-ethyl-3-(3-dimethylaminopropyl)carbodiimide hydrochloride] and Sulfo-NHS (N-hydroxysulfosuccinimide, Thermo Fisher Scientific) with w/w ratio (microtubules:EDC:Sulfo-NHS) of 1:1:2.2. After 20 min, 10 µg MCAK was added. The mixture was incubated for 1 h, before quenching with 2.5 M ammonium bicarbonate for 20 min on ice. The reaction mix was separated on a NuPAGE 4–12% Bis-Tris SDS-PAGE using MES running buffer and NuPAGE LDS sample buffer (ThermoFisher Scientific). The gel was stained with InstantBlue (Expedeon). Crosslinked samples (red box, Fig. 3C) were reduced, alkylated and trypsin digested following standard procedures (Maiolica et al., 2007). Crosslinked peptides were fractionated using SCX-StageTips following the published protocol for linear peptides and desalted using C18 StageTips (Rappsilber et al., 2007).

### Mass spectrometry

Peptides were analyzed on LTQ Orbitrap Velos (Thermo Fisher Scientific) coupled with a Dionex Ultimate 3000 RSLC nano system. The column was packed into a spray emitter (75-μm inner diameter, 8-μm opening, 250-mm length; New Objectives) that was packed with C18 material (ReproSil-Pur C18-AQ 3 μm; Dr Maisch GmbH, Ammerbuch-Entringen, Germany) using an air pressure pump (Proxeon Biosystems). Mobile phase A consisted of water and 0.1% formic acid. Mobile phase B consisted of 80% acetonitrile (CAN) and 0.1% formic acid. Peptides were loaded onto the column with 2% B at a 500 nl/min flow rate and eluted at a 300 nl/min flow rate with two gradients: a linear increase from 2% B to 40% B in 150 min; then an increase from 40% to 95% B in 11 min. The eluted peptides were directly sprayed into the mass spectrometer.

Peptides were analyzed using the following strategy: both MS spectra and MS2 spectra were acquired in the Orbitrap. FTMS spectra were recorded at 100,000 resolutions. The eight highest intensity peaks with a charge state of three or higher were selected in each cycle for iontrap fragmentation. The fragments were produced using CID with 35% normalized collision energy and detected by the Orbitrap at 7500 resolution. Dynamic exclusion was set to 90 s and the repeat count was 1. The mass spectrometric raw files were processed into peak lists using MaxQuant (version 1.5.3.30) (Cox and Mann, 2008), and crosslinked peptides were matched to spectra using Xi software (version 1.6.683). Search parameters were MS accuracy, 6 ppm; MS/MS accuracy, 20 ppm; enzyme, trypsin; crosslinker, BS3 (including BS3 modification); maximum missed cleavages, 4; fixed modification, carbamidomethylation on cysteine; variable modifications, oxidation on methionine; crosslinkable amino acids, N-terminus, lysine, serine, tyrosine and threonine; fragments, b and y ions with loss of H_2_O, NH_3_ and CH_3_SOH. The false discovery rate (FDR) was estimated using XiFDR on 5% residue level (Fischer and Rappsilber, 2017).

Data are available via ProteomeXchange with identifier PXD008215.

To compare MCAK and MCAK_S6E_ quantitatively, a workflow (Müller et al., 2018) that integrated the MaxQuant (v1.5.3.30) (Cox and Mann, 2008) for spectra pre-processing, Xi (v1.6.743, https://github.com/Rappsilber-Laboratory/XiSearch) for crosslinked peptide identification and Skyline (v4.1.0.11717) (MacLean et al., 2010) for MS1-based quantification was used. 130 unique residue pairs were quantified from 232 and 209 auto-validated unique residue pairs in MCAK and MCAK_S6E_ samples respectively across six analyses (three reaction replica, each reaction has two injection replica) using Orbitrap Fusion Lumos (Thermo Fisher Scientific) with a ‘high/high’ acquisition strategy. The peptide separation was carried out on an EASY-Spray column (50 cm×75 μm i.d., PepMap C_18_, 2 μm particles, 100 Å pore size, Thermo Fisher Scientific). Mobile phase A consisted of water and 0.1% v/v formic acid. Mobile phase B consisted of 80% v/v acetonitrile and 0.1% v/v formic acid. Peptides were loaded at a flow rate of 0.3 μl/min and eluted at 0.2 μl/min using a linear gradient going from 2% mobile phase B to 40% mobile phase B over 120 min, followed by a linear increase from 40% to 95% mobile phase B in 11 min. The eluted peptides were directly introduced into the mass spectrometer. MS data were acquired in the data-dependent mode with 3 s acquisition cycle. A precursor spectrum was recorded in the Orbitrap with a resolution of 120,000. The ions with a precursor charge state between 3+ and 8+ were isolated with a window size of 1.6 *m*/*z* and fragmented using high-energy collision dissociation (HCD) with collision energy 30. The fragmentation spectra were recorded in the Orbitrap with a resolution of 15,000. Dynamic exclusion was enabled with single repeat count and 60 s exclusion duration. The total peak area of 38 linear peptides has been used for run to run normalization. The peak area of each crosslinked residue pairs were calculated from the median value of six analyses. The data has been analysis with Perseus (v1.5.1.6) for *t*-test (Tyanova et al., 2016).

### Electron microscopy and image processing

Before use, the samples were freed of most aggregates by ultracentrifugation for 5 min at 90,000 rpm using a TLA-100 rotor and tabletop ultracentrifuge (Optima, Beckman Coulter). The MCAK samples were diluted from 0.025 to 0.1 mg ml^−1^ before being adsorbed onto a carbon-coated electron microscopy grid and negatively stained using uranyl formate 2%. The samples were examined using a Technai G2 200 kV equipped with a TVIPS F416 CMOS camera. Data collection was performed at a magnification of 50,000 with a pixel size of 2.13 Å per pixel and a dose of about 10 electrons per Å^2^.

Alignment and classification into class averages were performed using the Spider software (Frank et al., 1996). Particles were manually ‘windowed out’ using Boxer (Eman suite) into 135×135 pixels images. A resulting Spider stack of particles was then normalized against the background. A first step of reference-free alignment and classification was performed from which a selected set of class averages was chosen as new references for multi-reference alignment and classification. Several rounds of multi-reference alignment and classification were then performed, and new references were selected from the class averages until no further improvement was obtained. For MCAK and MCAK–GFP samples, 1039 and 2800 particles were, respectively, selected.

### Generation of a MCAK–:tubulin model

The structures of tubulin–stathmin–tubulin tyrosine ligase, tubulin–MCAK and tubulin–Klp10A were superposed using Pymol (PDB 4IIJ, 5MIO and 6B0C, respectively) (Gigant et al., 2013; Tan et al., 2008; Wang et al., 2015).

## Supplementary Material

Supplementary information
