# Supplementary material for: The depolymerase activity of MCAK shows a graded response to Aurora B kinase phosphorylation through allosteric regulation
Source: J Cell Sci. 2019 Jan 14;132(4):jcs228353. doi: 10.1242/jcs.228353 (PMC6398471; doi:10.1242/jcs.228353)
Supplement: Supplementary information [file joces-132-228353-s1.pdf]

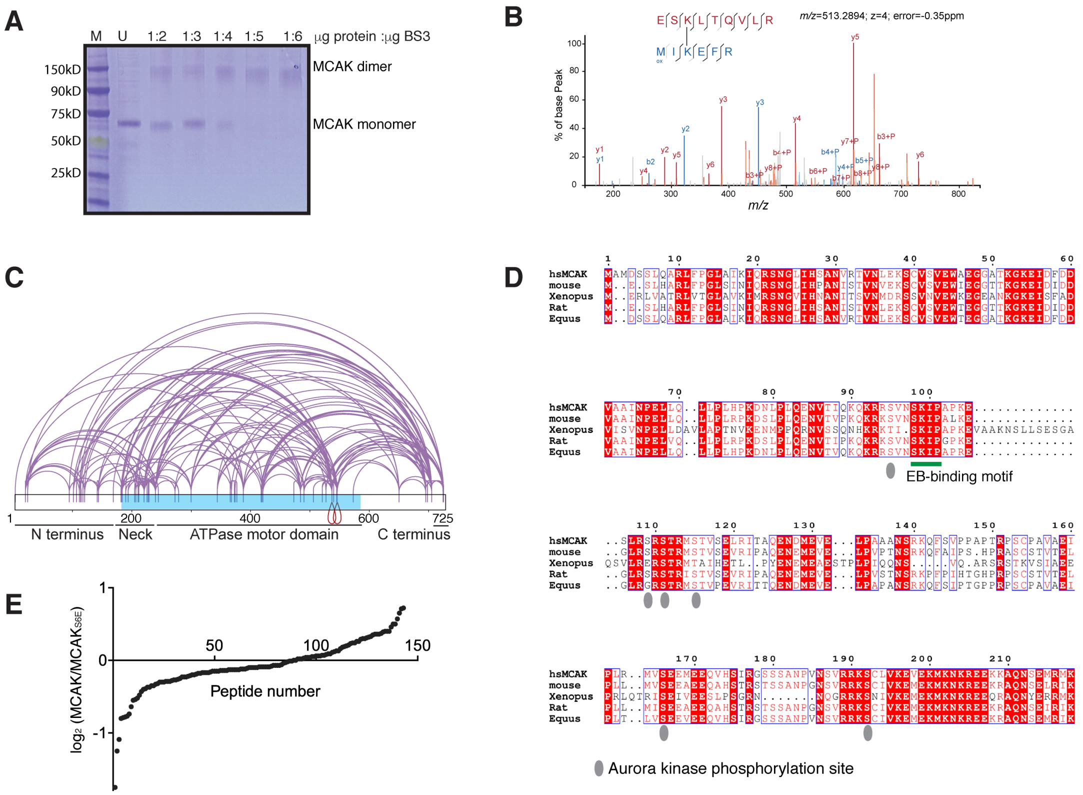

Figure S1. (A) SDS-PAGE coomassie-stained gel showing MCAK cross-linking with increasing amounts of BS3 cross-linker. U: uncross-linked. (B) Fragmentation spectrum of a cross-linked peptide pair that includes a link between Lys543 and Lys237. MS2 peaks supporting peptide sequence $ESK_{543}LTQVLR$ are annotated in red, and those supporting $MIK_{237}EFR$ are annotated in blue. (C) Cross-link pattern of MCAK using 5% FDR cut off. (D) Sequence alignment of MCAK for human, mouse, Xenopus, rat and horse species, highlighting conserved residues in red. Aurora B phosphorylated residues and the EB-binding motif are indicated. (E) Fold change of log_2 ratio for $MCAK/MCAK_{S6E}$ peptides.

Journal of Cell Science • Supplementary information

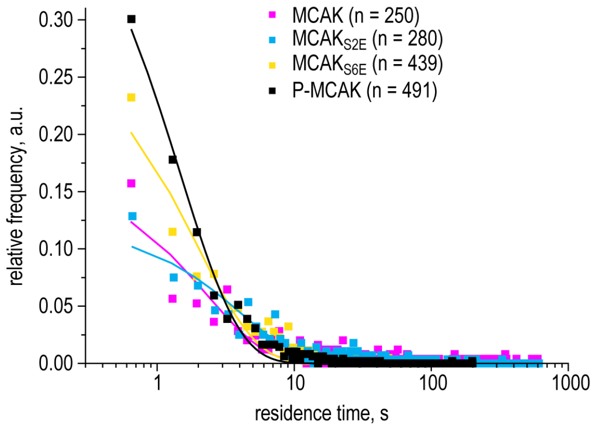

Figure S2. Distributions of residence times for MCAK-GFP (pink), MCAK$_{S2E}$–GFP (blue), MCAK$_{S6E}$-GFP (yellow) and Aurora B-phosphorylated MCAK-GFP (black) in 32 mM K-Pipes fitted with single exponential curves.
